# Efficacy of digital technology-based interventions for reducing caregiver burden and stress: a systematic review and meta-analysis

**DOI:** 10.3389/fdgth.2025.1636084

**Published:** 2025-11-06

**Authors:** Maria José Lumini, Daniela França, Maria Rui Sousa, Fátima Araújo, Carla Cardoso, Marta Sá, Mafalda Lopes, Maria José Peixoto, Teresa Martins

**Affiliations:** 1 Escola Superior de Enfermagem da Universidade do Porto, Porto, Portugal; 2 CINTESIS: RISE-Health, Porto, Portugal; 3 Escola Superior de Saúde Santa Maria, Porto, Portugal; 4 Instituto Português de Oncologia, Porto, Portugal; 5 Unidade Local de Saúde Póvoa do Varzim/Vila do Conde, Póvoa do Varzim, Portugal

**Keywords:** aging, informal care, caregiving burden, digital health interventions, systematic review

## Abstract

**Background:**

Demographic aging and increasing dependency associated with chronic diseases have intensified the caregiving responsibilities of family members, often leading to significant burden and stress. Digital technology-based interventions have emerged as promising strategies to support family caregivers, yet their effectiveness remains inconsistent across studies.

**Method:**

A systematic review and meta-analysis was conducted following JBI methodology and PRISMA guidelines. Literature searches were performed in CINAHL Complete, MEDLINE Complete, Scopus, and Web of Science (August 2024, updated September 2025). Studies were included if they involved family caregivers aged ≥18 years supporting individuals with functional dependency, implemented technology-based interventions, and employed experimental designs. Two independent reviewers conducted screening, data extraction, and quality assessment. Meta-analyses were performed to calculate standardized effect sizes (Cohen's d) for caregiver burden, stress, and quality of life outcomes.

**Results:**

Sixteen studies comprising 2,716 caregivers were included, predominantly randomized controlled trials. Interventions utilized diverse digital modalities including mobile applications, websites, telemonitoring, and tele-coaching, with most delivered by nurses. Meta-analysis revealed significant short-term reductions in caregiver burden (d = −0.65, 95% CI: −1.00 to −0.30, *p* < 0.01) and stress (d = −0.62, 95% CI: −0.81 to −0.43, *p* < 0.01). However, heterogeneity was substantial for burden (I^2^ = 75%) and effects on quality of life were non-significant with very high variability (I^2^ = 92%). Long-term effectiveness could not be determined due to limited follow-up data.

**Conclusion:**

Digital technology-based interventions demonstrate moderate effectiveness in reducing caregiver burden and stress in the short term. However, considerable variability in outcomes suggests that effectiveness is influenced by intervention characteristics, delivery modalities, and contextual factors. Future research should focus to strengthen the consistency of the findings, including subgroup analyses by type of intervention and evaluation of their long-term effects.

**Systematic Review Registration:**

PROSPERO CRD42024574765.

## 1 Background

In Europe, the demographic shift characterised by a persistently low birth rates and increased life expectancy is leading to an ageing population (1). This transformation, combined with the increasing dependence associated with chronic diseases, poses considerable challenges to healthcare systems and families' social and economic structures (2, 3).

Family caregivers, often without formal healthcare training, face complex and demanding responsibilities previously undertaken by professionals, increasing the risk of burden and stress (4, 5). The literature shows that this experience can lead to adverse consequences for caregivers' well-being and quality of life (6–8). Therefore, promoting effective interventions that mitigate stress and burden, while enhancing the well-being of family caregivers is a public health priority.

In recent years, technology-based interventions have emerged as promising strategies to support caregivers. E-learning platforms, mobile applications, telemonitoring, and augmented reality offer innovative approaches that provide information, training, and ongoing, personalised support (9–11).

The evidence suggests that technology-based interventions can effectively reduce caregiver burden and stress (12–17). Recent systematic reviews with meta-analysis confirmed this effectiveness among informal carers of older adults (12, 17). The specific type of intervention appears to modulate this effect (12–17), underscoring the importance of selecting an appropriate delivery modality that accounts for the strengths and limitations of each strategy (12, 13, 15). Although the overall findings are promising, results across studies are not entirely consistent, indicating variability in outcomes depending on context, intervention type, and implementation (12, 17).

In this context, it is essential to critically synthesise the available evidence on the effectiveness of technology-based interventions for family caregivers. The present study aims to conduct a systematic review of the effectiveness of interventions that utilise technology-based approaches to reduce caregiver burden and stress or to enhance the quality of life and well-being of family caregivers, following the research question: Is there evidence that technology-based interventions decrease the burden of family caregivers? By analysing different technological approaches and related outcomes, this study seeks to identify promising practices and contribute to the development of more efficient and accessible strategies to support family caregivers.

## 2 Methods

### 2.1 Design

The proposed systematic review will be conducted in accordance with JBI methodology for systematic reviews of effectiveness (18) and reported in line with the Preferred Reporting Items for Systematic Reviews and Meta-Analyses (PRISMA) guidelines (19). The review protocol was registered in the PROSPERO database (https://www.crd.york.ac.uk/PROSPERO/view/CRD42024574765).

Table 1 summarises the inclusion criteria for this review following the PICOD acronym. Additionally, studies had to be published in Portuguese, English, or Spanish, be available in full text and were not subject to any publication date restrictions.

**Table 1:** Selection inclusion and exclusion criteria.

Selection criteria	Inclusion criteria	Exclusion criteria
Participants	Family caregivers aged 18 years and over who support individuals with functional dependency in activities of daily living	Family caregivers aged less than 18 years or formal caregivers.
Intervention	Technology-based interventions aimed at reducing caregiver burden and stress, as well as enhancing caregivers’ well-being. These interventions include digital resources such as remote monitoring systems, telehealth services, mobile applications, and online educational platforms that provide continuous support and guidance. Such interventions may be self-directed or facilitated by healthcare professionals.	Interventions relying exclusively on telephone communication
Comparison	Any comparison interventions or control groups	
Primary Outcome	Burden or Stress	Studies that did not assess caregiver burden or stress
Secondary Outcome	Well-being or Quality of life	
Study Design	Randomized Control Trials (RCTs) and other experimental studies	Study designs not specified in the inclusion criteria, such as observational or qualitative studies

### 2.2 Search methods

The search and identification of studies in the databases took place in August 2024 and an updating September 2025. The literature search was conducted in the following databases: CINAHL Complete, MEDLINE Complete (*via* EBSCOhost), Scopus, and Web of Science. Search terms were derived from the elements of the PICOD framework. In databases using controlled vocabularies (CINAHL and MEDLINE), Medical Subject Headings (MeSH) and CINAHL Headings were employed to increase specificity. The search strategies were adapted to the requirements of each database and could be consulted in supplementary material.

### 2.3 Data extraction

The results from the bibliographic search were imported into Rayyan (20) to remove duplicates and conduct an initial screening of titles and abstracts based on predefined inclusion criteria. Two authors independently analysed and extracted the data, resolving disagreements through discussion or, if necessary, with a third reviewer, following JBI methodological recommendations (18). Selected studies then underwent full-text review. A data extraction form in Microsoft Excel was used to collect information on study title, authors/year, country, objectives, sample, design, instruments, intervention type or content, effectiveness results, effect size, and study limitations.

### 2.4 Quality assessment

The methodological quality of the included studies was assessed using the JBI Critical Appraisal Checklist for Randomized Controlled Trials (21) and the Checklist for Quasi-Experimental Studies (22). Two independent reviewers conducted the quality assessments for ensure the methodological quality of the articles. Potential disagreements were solved by a third reviewer.

### 2.5 Statistical analysis

The meta-analysis was conducted using IBM SPSS (version 30.0). Studies providing sufficient data, including means, standard deviations, and sample sizes, were included. Extracted data also encompassed effect sizes or sufficient information to calculate them; when such data were unavailable, the study authors were contacted. As the included studies employed different caregiver burden and stress scales, standardised effect sizes (Cohen's d) with 95% confidence intervals were calculated to enable comparability across studies. Negative values indicated a reduction in caregiver burden or stress in the intervention group compared with controls or baseline measurements. Heterogeneity was assessed using Cochran's *Q* test and the I^2^ statistic. A random-effects model was applied to account for potential heterogeneity across studies. Forest plots were generated to visualise study weights and the consistency of effects. Sensitivity analyses were conducted by excluding studies at high risk of bias. The findings of studies that were not comparable and could not be included in the statistical pooling were presented narratively.

## 3 Results

### 3.1 Search results

**Figure 1:**
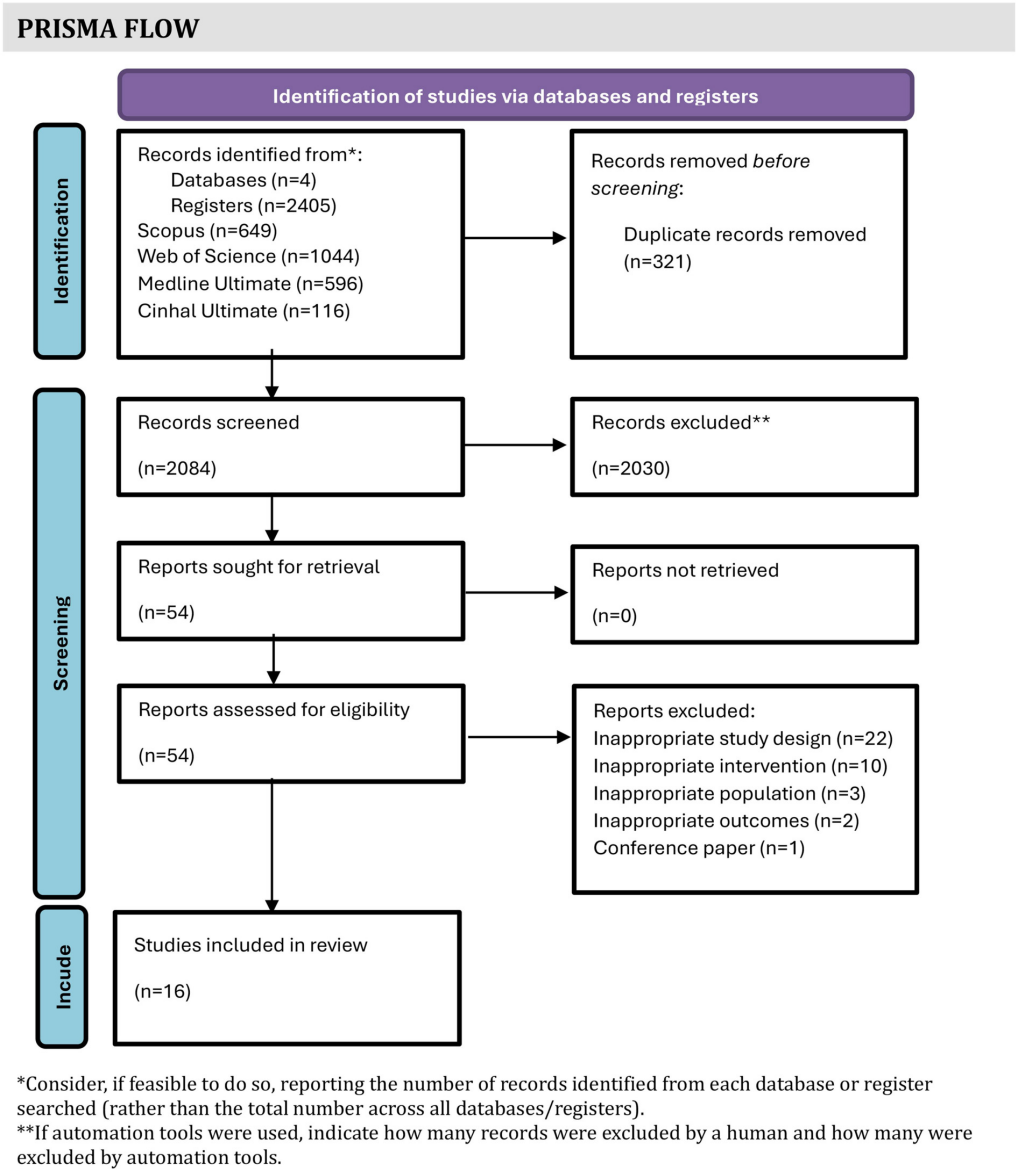
PRISMA flow diagram.

A total of 2,405 articles were identified. After excluding duplicates, the remaining 2,084 records were screened by title and abstract. Finally, 54 full-text reports for further assessment were retrieved. After evaluating their eligibility, 16 studies met the inclusion criteria. A flow diagram is presented in Figure 1 (19).

### 3.2 Study characteristics

The characteristics of the included studies and their participant populations are summarised in Table 2. Of the 16 included studies, most of them were published in the last five years, were conducted in the USA and employed RCT designs.

**Table 2:** Characteristics of the studies and participants.

Author/year/country	Objectives	Target population
Rahimi et al. 2025 Iran	Evaluate the impact of Mehrpishegan's web-based intervention on depression, anxiety, and stress levels among informal primary caregivers of older adults.	Caregivers of elderly people
Kabotari et al. 2025 Iran	Explore the effects of an online home care training program on caregiver burden in those caring for patients with multiple sclerosis.	Caregivers of patients with multiple sclerosis
Ganefianty et al. 2024 Indonesia	Assess the effectiveness of a mobile health (m-health) transitional care intervention to reduce the stress and burden of caregivers and reduce readmission.	Caregivers of patients with traumatic brain injury
Minaei-Moghadam et al. 2024 Iran	Investigate the effectiveness of a supportive care program via a smartphone application on the quality of life and care burden among family caregivers.	Caregivers of patients with major depressive disorder.
Riegel et al. 2024 USA	Test the efficacy of a virtual health coaching intervention, compared with health information alone, on the self-care, stress, coping, and health status of caregivers.	Caregivers of adults with chronic heart failure
Douglas et al. 2023 USA	Compare the effectiveness of a remotely delivered intervention with 2 arms (i.e., website and tele-coaching vs. website only) on reduction of depression, anxiety, stress, and distress.	Caregivers of people with multiple sclerosis.
Tinoco-Camarena, et al. 2023 Spain	Evaluate the effectiveness of an online nursing intervention to increase Positive Mental Health and reduce burden.	Caregivers of patients with complex chronic conditions
Hepburn et al. 2022 USA	Evaluate the effectiveness of the Tele-Savvy online psychoeducation programme for family caregivers. The programme aimed to provide caregivers with essential support and resources to enhance their well-being and improve their caregiving experience, ultimately addressing the challenges they face in their roles.	Family caregivers of persons living with dementia
Ferré-Grau et al. 2021 Spain	Evaluate the effectiveness of a smartphone app–based intervention program to increase positive mental health for nonprofessional caregivers.	Caregivers of people with chronic diseases
Namjoo et al. 2021 Iran	Determine the impact of telenursing on the short-term caregiver burden of patients with heart failure discharged from hospitals.	Caregiver burden among Iranian patients with heart failure
Fuller-Tyszkiewicz et al. 2020 Australia	Evaluate the effectiveness of a self-guided mobile app–based psychological intervention for people providing care to family or friends with a physical or mental disability.	Caregivers of people with a physical or mental disability
Possin et al. 2019 USA	Determine whether the Care Ecosystem effectively improves outcomes important to persons with dementia, their caregivers, and payers beyond those achieved with usual care.	Caregivers of persons with dementia
Meichsner et al. 2019 Germany	Evaluate the efficacy of an internet-delivered cognitive-behavioural intervention for caregivers and examine acceptance of program characteristics.	Caregivers of Persons with dementia
Applebaum et al. 2018 USA	Evaluated the feasibility, acceptability, and preliminary effects of the web-based program: Care for the Cancer Caregiver Workshop	Caregivers of persons with cancer
Easom et al. 2018 USA	Evaluate the effectiveness of the Operation Family Caregiver (OFC) program, which provided problem-solving training (PST) to caregivers.	Military caregivers
Chiang et al. 2012 Taiwan	Evaluate the effectiveness of traditional nurse-led care combining discharge planning and telehealth care on family carer burden, stress management and family function of family carers compared to those receiving only traditional discharge planning.	Family caregivers of heart failure patients

### 3.3 Characteristics of the participants

The studies comprised 2,716 caregivers of patients with a variety of diseases or health conditions (Table 2). Some studies were targeted at caregivers of patients with dementia (23–25), chronic heart failure (26–28), chronic health conditions (29, 30), traumatic brain injury (31), major depressive disorders (32), multiple sclerosis (33, 34), cancer (35), physical and mental disabilities (36, 37) elderly (38), and military with post-traumatic injuries (36). Sample sizes of each study ranged from 27 to 780 participants. Most of the caregivers were spouses or partners (23–28, 31–36), adult children caring for their parents (29, 30, 38) and parents caring for their children (37).

In all studies, most participants were female except one (27). Caregivers' educational attainment ranged from secondary level to graduate level.

### 3.4 Characteristics of the interventions

The key characteristics and content of the interventions are summarised and presented in Table 3.

**Table 3:** Characteristics of the interventions.

Author/Year Design	Content of intervention	Delivery mode and learning modalities	Time per session and frequency and duration	Intervenors	Sample
Rahimi et al. 2025 RCT	The intervention provided caregivers with tailored educational resources, including text, audio, and video materials developed with input from specialists. Grounded in Schema Therapy, the programme emphasised peer support and vicarious learning, addressing common maladaptive schemas while offering emotional and social support, opportunities for peer sharing, coping strategies, and self-care guidance.	An online platform with combined multimedia resources, three asynchronous forums, and an online chat room. For the first three months, the online chat was led weekly by a psychologist. For the following three months, the online chat was led by the participants, promoting peer support.	Sessions lasted 60–90 min, held weekly for the first three months with psychologist-led discussions, followed by independent online peer sessions, totalling 18–24 sessions.	Psychologist	*N* = 165 (IG = 83, CG = 80) The CG accessed only basic website features, without the interactive or educational components available to the intervention group.
Kabotari et al., 2025 RCT	The programme consisted of 14 video clips, covering managing multiple sclerosis (MS). The content included topics such as movement disorders, gastrointestinal symptoms, urinary issues, sexual dysfunction, swallowing and speech difficulties, pain management, fatigue, pharmacotherapy care, and nutritional guidance.	The online course was delivered via WhatsApp, with training materials provided in multiple formats, including video clips, audio recordings, written documents, and images. Each module was complemented by two follow-up phone calls to caregivers: the first to confirm receipt and access to the material, and the second, conducted four days later, to assess understanding and application with a checklist.	The program lasted for 8 weeks, with educational content sent weekly.	Nurses	*N* = 80 (*n* = 40 in each group) CG: received the questionnaires at the same intervals as the IG, with the educational content provided at the end of the study.
Ganefianty et al. 2024 RCT	Information regarding how to 1) treat patients with traumatic brain injury at home, including craniotomy wound care and how to provide nutrition; 2) recognize signs of infection in craniotomy wounds; 3) recognize emergencies at home; 4) manage stress; and 5) arrange a schedule for the care of the patients.	m-health app with chat, phone support, monitoring, and follow-up (online chat service for communication between nurses and caregivers, contact phone number for assistance); complemented by face-to-face education before hospital discharge using flip charts and demonstration of caring skills.	Weekly monitoring via phone for one month after discharge.	Nurses	*N* = 74 (*n* = 37 in each group). CG: Standard care per hospital procedures
Minaei-Moghadam et al. 2024 RCT	Nutrition, medications, psychotherapies, sleep hygiene, regulation of activities, the definition of depression and its related disorders, symptoms of major depressive disorder, and suicidal thoughts, related care, diagnostic measures, communication strategies, and electroconvulsive therapy and related care.	Smartphone app with multimedia content (audio, videos, and photos), medication time reminders, and chat with a nurse at specific times; complemented by face-to-face training with multimedia content and the ability to communicate, before the app use.	Weekly app use and phone calls for one month.	Nurses	*N* = 60 (IG = 29; CG = 31) CG: Standard interventions with face-to-face training (10–15 min) focused on medication use, important side effects, and weekly phone support.
Riegel et al. 2024 RCT	Caregiving demand, stress management, self-care, sleep, thought distortions, automatic thoughts, relaxation techniques, and confidence.	Virtual intervention with a Samsung tablet with mobile connectivity, vetted websites, and video/audio communication; complemented by online video interventions, a quarterly emailed newsletter (featuring recipes, caregiver tips, local events), and synchronous coaching sessions (addressing caregiver concerns).	≥30-minute weekly website review, monthly reminders and 10 synchronous coaching sessions over 6 months.	Health coaches (nurses or public health nurses)	*N* = 250 (*n* = 125 in each group). CG: No access to the synchronous coaching sessions but provided tablets and website content.
Douglas et al. 2023 RCT	Provided information on multiple sclerosis, caregiving strategies, and self-care resources. Sessions covered caregivers’ needs, care strategies, self-care, and decision-making. Website use was not specifically encouraged.	Tele-coaching sessions (videoconference or telephone) combined with a curated website; complemented by psycho-educational intervention with two approaches: one combining a curated website with tele-coaching sessions, and another utilizing only the website.	Total duration of 4 months, during which coaching group participants received four personalized coaching sessions spread over 6 weeks, each lasting 35 to 40 min.	Independent social workers	*N* = 151 (IG = 75; CG *n* = 76). CG: Received access to the website but no coaching sessions.
Tinoco-Camarena, et al. 2023 RCT	Addressing caregivers’ problems, difficulties, and feelings through dialogue circles to decrease caregiving burden and enhance positive mental health.	Online intervention via the Zoom platform; complemented by dialogue circles to decrease caregiving burden and increase positive mental health, including group reflection, social support, facilitated discussions, and sharing beneficial strategies.	Each session lasted approximately 90 min, with a 15-day interval between each, in a total of 3 dialogue circles.	Nurse	*N* = 86 (*n* = 43 in each group). CG: Received routine care and referral to social work for information on available assistance.
Hepburn et al. 2022 RCT	Knowledge about dementia, caregiving skills, self-care strategies (including strategies to help caregivers manage the frequent negative emotions), active learning exercises, peer support, and mindful self-care.	Online synchronous and asynchronous platform (Tele-Savvy); complemented by online psychoeducation, including synchronous learning via weekly Zoom sessions with discussions, exercises, and coaching, and asynchronous learning with daily video lessons on dementia caregiving topics.	Delivered over 43 days, 7 weekly synchronous sessions (75–90 min), and 36 brief asynchronous video lessons. Daily video lessons and six additional mindful self-care lessons provided between sessions.	Facilitator (not specified the type of professionals) with prior experience leading the in-person Savvy program	*N* = 261 (3 arms): 1) Active online group (*n* = 96); 2) Attention control (*n* = 111); 3) Usual care/waitlist (*n* = 54). ACG joined “Healthy Living” (similar to Tele-Savvy but without emotional/psychological focus) or continued usual caregiving without structured training.
Ferré-Grau et al. 2021 RCT	Content included daily activities based on 10 recommendations to promote positive mental health. Includes gamification, motivational quotes, coaching messages, and daily check-ins (39)	Mobile App (TIVA); complemented by structured activities and resources to promote positive mental health, self-paced learning, and flexibility in engagement. Caregivers had autonomy to manage their time and choose when to engage with the app's activities, with 2 activities created for each recommendation.	Each participant used the app for 28 days with daily activity, from Monday to Friday.	Nurses	*N* = 113 (IG = 56; CG = 57) CG: Received standard care from nursing staff at primary health care centres, with psychological intervention referrals.
Namjoo et al. 2021 RCT	Content was delivered in 15 separate parts (not specified) with training photos and videos. The material was designed to be simple, comprehensible, and loaded with relevant information.	Delivered through social media (not identified); complemented by educational material for patients and their caregivers, with encouragement to study and engage to ensure comprehension and adherence to the care plan.	Delivered every other day for one month.	Researchers (nurses and physicians)	*N* = 100 (*n* = 50 in each group) CG: Received only routine care and training during hospital discharge.
Fuller-Tyszkiewicz et al. 2020 RCT	Five modules: 1) stress; 2) values clarification and goal setting; 3) mindfulness; 4) positive psychology and cognitive restructuring; 5) behavioural activation for meaningful activities.	Mobile app (StressLess); complemented by psychoeducation (through text, video, audio, and graphics) and interactive exercises.	App used for 5 weeks with weekly email or phone contact from the research team to enhance engagement.	Research team	*N* = 183 (IG = 73; CG = 110) CG: With an app (StressMonitor) for monitoring stress and well-being.
Possin et al. 2019 Pragmatic RCT	Education and support on dementia, management strategies, and resources for daily care.	Telephone and internet-based supportive care (Care Ecosystem); complemented by a personalised and structured interactive approach tailored to the specific needs of caregivers by videoconference and telephone calls.	The care team navigators made telephone calls or videoconferences approximately once a month over 12 months.	Care team navigators (supported by expert providers)	*N* = 780 (IG = 512; CG = 268) CG: Usual care, access to caregiver resources (FCAA, Area Agencies on Aging), and quarterly newsletters.
Meichsner et al. 2019 RCT	Ten modules (Basic elements; Problem analysis, Psychoeducation; Strengthening problem-solving abilities; Changing dysfunctional cognitions, Increasing the use of informal and/or professional support; Coping with change, grief and loss; Self-care creating value-based activities; Stress management and emotion regulation strategies; Evaluation) and information on dementia, its progression, and its effects (40).	Online platform (Tele.TAnDem); complemented by a cognitive-behavioural therapy program with written modules, intervention, videos and images, personalized messages, and therapist responses. The program also incorporated elements of emotional support.	8-week internet intervention with 10 written modules.	Clinical psychologist	*N* = 27 (IG = 13; CG = 14) CG: Wait-list control group received written content about the disease and dementia care.
Applebaum et al. 2018 RCT	The introductory webcast provides an overview (legacy, choice, creativity, and connectedness) and discusses the concept of identity in caregiving. The subsequent four webcasts are dedicated to the four sources of meaning targeted by the meaning-centred psychotherapy programme. Each webcast involves responses to thought-provoking questions based on the meaning-centred psychotherapy cancer manual.	Web based program (Care for the Cancer Caregiver Workshop); complemented by self-administered webcasts, didactic components, therapeutic video clips, and interactive message boards, where participants could post responses to experiential exercises and interact with other participants and post responses.	Five webcasts completed within 14 weeks. Caregivers were helped with reminders every two weeks to complete webcasts and follow-up assessments.	Research team	*N* = 84 (*n* = 42 in each group) Wait-list CG: received usual care, including the American Cancer Society hotline and information on local and national resources.
Easom et al. 2018 Pre-post research	Problem-solving techniques, stress management, communication, self-care, and emotional support. Structured approach using D'Zurilla and Nezu's social problem-solving model.	The program was delivered via virtual platforms (e.g., Skype), face-to-face sessions, and phone calls. It included five key problem-solving steps (gathering facts, fostering optimism, goal setting, decision-making, and generating solutions), with access to resources through a secure website. The process involved building a rapport with a coach, brainstorming, role-playing, follow-up calls, and face-to-face sessions addressing new problems and self-care strategies.	Six-month period, weekly sessions.	Trained coaches (without more specification)	*N* = 370 Without a control group.
Chiang et al. 2012 Quasi-experimental	Knowledge of managing heart failure, recognising symptoms and understanding treatment plans (i.e., dietary therapy and limiting fluid intake).	Telehealth technology connecting caregivers to a central platform (National Taiwan University Hospital); complemented by a nurse-led transitional care program with continuous education, counselling, 24/7 nursing support, follow-up calls, and data monitoring by caregivers at home.	One-month post-hospital discharge.	Cardiac physicians and nurses	*N* = 60 (*n* = 30 in each group) CG: The 30 families who declined telehealth care received standard care.

#### 3.4.1 Intervention components, educational content, and delivery approaches

**Figure 2:**
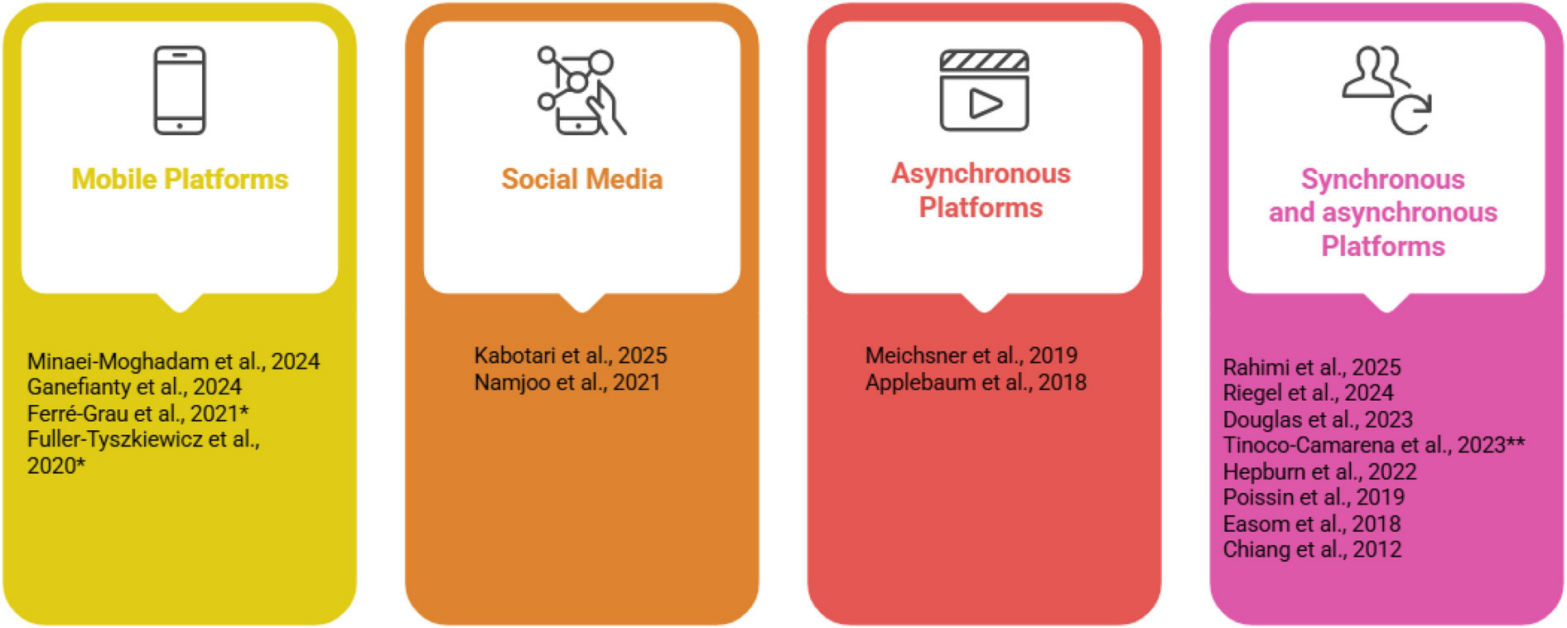
Interventions by delivery mode and learning modality. *without professional interaction; **only synchronous. Created using Napkin AI.

Except one article (29) all the others were focussed on training essential skills related to patient care. Additionally, some studies incorporated topics like stress management (23, 24, 28, 30–32, 35–37), problem-solving techniques (36), sleep hygiene (28, 32), communication strategies (32, 36), relaxation techniques (24, 28, 37), sharing experiences among peers (38), self-care (23, 28, 33, 36), resources for caregivers (23, 25, 30, 33, 38), positive mental health techniques (29, 30), and cognitive restructuring techniques (37). Caregivers had access to interactive resources and tools, allowing them to practice caregiving strategies in real-life scenarios while being provided support in dealing with negative emotions and developing self-care practices. Programmes also included personalised coaching and follow-up sessions, which helped address their specific challenges and encouraged peer interaction to foster shared learning and support. Table 3 summarises the content of the interventions. The interventions employed diverse digital delivery modes and learning modalities (Figure 2).

Several studies supplemented digital components with telephone follow-ups (25, 26, 31, 33, 36), online chat discussions (30, 32, 38), and tele-coaching (23, 25, 28, 33, 36).

The educational resources included flip charts, newsletters, written materials, audio recordings, videos, images, reflective exercises, lectures, and home practice activities. In four studies, the intervention was entirely self-managed without direct interaction with health professionals (27, 29, 35, 37). Each study employed a unique combination of these approaches (Table 3).

#### 3.4.2 Programme duration, frequency, intervenors, and control conditions

Most studies involved weekly interventions, with only one study including daily (29) or alternate-day interventions (27). One study mentioned 24 h nursing support (26). The overall duration of the intervention programmes ranged from one to six months (Table 3).

Of the interventions, six were delivered solely by nurses (28–32, 34). Others were facilitated by nurses and physicians (26, 27), psychologists (24, 38), social workers (33), research teams (35, 37), care teams navigators (25), coaches (36), and facilitators (23).

In three studies, the caregivers in the control group were placed on a waitlist and received the intervention following its delivery to the experimental group (23, 24, 35). In the other ten studies, the control groups received standard or usual care (25–32, 34). In one study, the control group was exposed to a different app that monitored their stress levels and well-being (37), while others provided website access without any coaching sessions (33) or online chat room (38). One study did not have a control group (36).

### 3.5 Caregivers' outcomes and measurements

The most frequently assessed outcome was caregiver burden. Table 4 summarises the outcome variables and measurements used across all the 16 studies. Several measures were used to assess caregiver stress, burden, quality of life, and well-being. Notably, the Zarit Burden Interview (ZBI) and the Caregiver Burden Inventory (CBI) were commonly employed. Quality of life was measured by the WHOQOL-BREF and SF36, and its mental health dimension was assessed through the Positive Mental Health Questionnaire. Stress levels were assessed using one subscale of the Depression Anxiety Stress Scale (DASS) and the Perceived Stress Scale (PSS). Well-being was assessed in only two studies using different instruments.

**Table 4:** Measures used and results.

Study	Instruments	Assessment moment	Results/efficacy
Rahimi et al. 2025	Stress: Depression, Anxiety, and Stress Scale (DASS)	Baseline (T0), 3 months (During the intervention T1) and 6 months (end of the intervention T2)	Stress A small reduction in stress in the IG compared with CG over time, but without statistically significant (mean difference =1.22, 95% CI: −3.37 to 0.92, *p* = 0.261; with a limited effect of the web-based intervention.
Kabotari et al. 2025	Burden: Zarit Burden Interview (ZBI)	Baseline (T0), 8 weeks (end of the programme T1), 4 weeks after intervention (T2)	Burden T0CG = 47.82 (± 5.04); *n* = 40 T0IG = 44.75 (±9.37); *n* = 40; *p* = 0.072 T1CG = 48.95 (±4.44); *n* = 40 T1IG = 41.45 (±10.68); *n* = 40; *p* = 0.01 T2CG = 45.75 (±7.71); *n* = 40 T2IG = 45.20 (±10.88); *n* = 40; *p* = 0.58
Ganefianty et al. 2024	Stress: The 20 items of Caregiver Stress Self-assessment Burden: The 12 items Zarit Burden Interview Short-Form	Baseline (T0), 2 weeks (T1), 1 month (end of the programme T2)	Stress T0CG = 29.38 (±4.5); *n* = 37 T0IG = 28.59 (±5.4); *n* = 3; *p* = 0.112 T1CG = 26.18 (±4.5); *n* = 37 T1IG = 22.84 (±3.1); *n* = 37; *p* < 0.001 T2CG = 23.24 (±6.3); *n* = 37 T2IG = 17.49 (±2.1); *n* = 37; *p* < 0.001 Burden T0CG = 22.11 (±3.0); *n* = 37 T0IG = 19.78 (±3.5); *n* = 37; *p* = 0.072 T1CG = 22.62 (±2.9); *n* = 37 T1IG = 15.03 (±2.1); *n* = 37; *p* < 0.001 T2CG = 21.59 (±3.1); *n* = 37 T2IG = 11.62 (±1.5); *n* = 37; *p* < 0.001
Minaei-Moghadam et al. 2024	Burden: 24 items Novak and Guest´s Caregiver Burden Inventory (CBI) Quality of life: 12 items WHOQOL-BREF	Baseline (T0), end of the programme (1 month-T1)	Burden T0CG = 87.8 (±11.2); *n* = 31 T0IG = 88.7 (±13.7); *n* = 30; *p* = 0.778 T1CG = 89.8 (±7.3); *n* = 29 T1IG = 76.1 (±8.8); *n* = 29; *p* < 0.001 Quality of life T0CG = 73.8 (±13.6); *n* = 31 T0IG = 70.9 (±8.3); *n* = 30; *p* = 0.320 T1CG = 70.9 (±11.5); *n* = 29 T1IG = 83.2 (±7.1); *n* = 29; *p* < 0.001
Riegel et al. 2024	Stress: Perceived Stress Scale Quality of life: SF36 Short-form Scale	Baseline (T0), end of the programme (6 months-T1)	Stress T0CG = 26.5 (±7.7); *n* = 125 T0IG = 25.9 (±7.5); *n* = 123; *p* = 0.55 T1CG = 25.20 (±8.71); *n* = 100 T1IG = 19.73 (±6.97); *n* = 92; d = −0.59; *p* < 0.0001 Quality of life (mental health subscale) T0C = 41.1 (±12.2); *n* = 123 T0I = 43.2 (±12.3); *n* = 120; *p* = 0.18 T1CG = 42.73 (±12.10); *n* = 99 T1IG = 48.18 (±10.43); *n* = 92; d = 0.30; *p* = 0.04
Douglas et al., 2023	Stress: Depression, Anxiety, and Stress Scale (DASS)	Baseline, 6 weeks, end of the programme (4 months)	The IG showed a significantly greater reduction in stress than the website-only CG. A higher proportion of participants achieved meaningful decreases in DASS Stress scores, and fewer experienced worsening stress levels. Statistical analysis confirmed a significant group-by-time interaction indicating that the website plus coaching intervention was more effective in reducing emotional distress (*p* = 0.037), and stress (*p* = 0.047).
Tinoco-Camarena et al. 2023	Burden: 7 items Zarit Burden Interview Quality of life - Mental health: 39 items Positive Mental Health Questionnaire	Baseline (T0); end of the programme (1month T1)	Burden (Median, interquartile range) T0CG = 22 (20, 25); *n* = 43 T0IG = 26 (24, 28); *n* = 43; *p* = 0.01 T1CG = 25 (22, 27); *n* = 43 T1IG = 20 (18,24); *n* = 37; *p* < 0.001 Mental health T0CG = 93 (90.5, 96.5); *n* = 43 T0IG = 91 (89, 95.5); *n* = 43; *p* = 0.467 T1CG = 89 (83,93); *n* = 43 T1IG = 112 (105.5, 118); *n* = 49; *p* < 0.01
Hepburn et al. 2022	Burden: 22 items Zarit Burden Inventory Stress: 14 items Perceived Stress Scale	Baseline (T0), 3 months (T1), 6 months (T2-Follow-up)	Burden T0ACG = 35.78 (±14.66); *n* = 111 T0WLCG = 35.94 (±15.99); *n* = 54 T0IG = 37.34 (±13.77); *n* = 96 T1ACG = 36.28 (±13.49); *n* = 83 T1WLCG = 37.82 (±15.80); *n* = 43 T1IG = 35.26 (±13.03); *n* = 69 T2ACG = 35.91 (±12.96); *n* = 80 T2WLCG = 37.48 (±17.31); *n* = 45 T2IG = 35.54 (±13.63); *n* = 72 Stress T0ACG = 22.66 (±8.21); *n* = 111 T0WLCG = 21.73 (±7.71); *n* = 54 T0IG = 23.50 (±8.93); *n* = 96 T1ACG = 21.24 (±7.63); *n* = 83 T1WLCG = 23.86 (±6.74); *n* = 43 T1IG = 20.77 (±6.93); *n* = 71 T2ACG = 22.14 (±8.08); *n* = 80 T2WLCG = 23.50 (±7.36); *n* = 46 T2IG = 20.72 (±7.10); *n* = 72
Ferré-Grau et al. 2021	Burden: 7 items Zarit Caregiver Burden short form Quality of life - Mental health: 39 items Positive Mental Health Questionnaire (PMHQ)	Baseline (T0), 1 month (end of the programme T1), 3 months (T2)	Burden T0CG = 19.77 (±5.38); *n* = 43 T0IG = 18.80 (±5.64); *n* = 49; *p* = 0.43 T1CG = 20.56 (±5.24); *n* = 43 T1IG = 18.29 (±5.34); *n* = 49; *p* = 0.04 T2CG = 20.70 (±5.44); *n* = 43 T2IG = 17.69 (±5.52); *n* = 49; *p* = 0.01 Positive Mental Health T0CG = 120.10 (±20.32); *n* = 57 T0IG = 98.60 (±10.96); *n* = 56; *p* < 0.001 T1CG = 118.94 (±20.05); *n* = 43 T1IG = 101.55 (±14.70); *n* = 49; *p* < 0.001 T2CG = 121.68 (±19.52); *n* = 43 T2IG = 114.41 (±20.30); *n* = 49; *p* = 0,08
Namjoo et al. 2021	Burden: 22 items Caregiver Burden Scale by Elmstahl et al.	Baseline (T0), end of the programme (1month T1)	Burden T0CG = 37.26 (±13.27); *n* = 50 T0IG = 35.56 (±19.84); *n* = 50; *p* = 0.62 T1CG = 34.58 (±19.84); *n* = 50 T1IG = 24.28 (±11.22); *n* = 50; *p* = 0.001
Fuller-Tyszkiewicz et al. 2020	Stress: 21 items Depression Anxiety Stress Sale Well-being: 21 items Personal Well-being Index (PWI)	Baseline (T0), end of the programme (5 weeks T1)	Stress T0CG = 18.82 (±7.98); *n* = 110 T0IG = 17.03 (±7.88); *n* = 73; *p* = 0.14 T1CG = 18.94 (±9.03); *n* = 110 T1IG = 14.72 (±7.49); *n* = 73; *p* = 0.001 Well-being T0CG = 58.02 (±15.18); *n* = 110 T0IG = 55.73 (±16.15); *n* = 73; *p* = 0.33 T1CG = 54.72 (±17.06); *n* = 110 T1I = 57.98 (±17.54); *n* = 73; *p* = 0.21
Possin et al. 2019	Burden: 12-ItemZarit Burden Interview	Baseline, 6 months, 12 months (end of the programme)	Caregiver burden declined more in the Care Ecosystem group than in the CG at 12 months (*β*=−1.90; 95% CI, −3.89 to −0.08; *p* = 0.046); the 6-month treatment effect was also statistically significant (β=−1.51; 95% CI −2.63 to −0.39; *p* = 0.008).
Meichsner et al. 2019	Burden care was measured with a visual analogue scale ranging from 0 = I do not feel burdened to 100 = I feel highly burdened Emotional well-being measured with a visual analogue scale ranging from 0 = I am in a very dark mood to 100 = I am in a very joyful mood	Baseline (T0), end of the programme (8 weeks; T1), 6 months (Follow-upT2)	Burden T0CG = 76.00 (±22.02); *n* = 18 T0IG = 76.11 (±15.60); *n* = 19; *p* = 0.97 T1CG = 79.40 (±15.40); *n* = 15 T1IG = 72.33 (±18.70); *n* = 15; *p* = 0.27 T2CG = 61.14 (±26.44); *n* = 14 T2IG = 73.08 (±17.88); *n* = 13; *p* = 0.19 Emotional well-being significantly improved over time (β=0.96, *p* = 0.023), with 75.7% of participants showing positive changes. Baseline well-being predicted well-being during the intervention (β=0.47, *p* = 0.002), but no significant interaction was observed between time and initial well-being.
Applebaum et al. 2018	Burden: 24 items Caregiver Reaction	Baseline (T0), 15 weeks (end of the programme T1), 25 weeks (Follow-up T2)	Baseline T0IG M = 82.24 (±11.20); *n* = 42 T0WCG M = 80.21 (±10.81); *n* = 42; *p* = 0.40 T1IG M = 82.26 (±11.29); *n* = 32 T1CG M = 79.47 (±12.74); *n* = 34; *p* = 0.35 T2IG M = 79.49 (±9.49); *n* = 20 T2CG M = 79.42 (±12.25); *n* = 31; *p* = 0.98
Easom et al. 2018	Burden: 22 items Zarit Burden Scale	Baseline, End of the programme (6 months)	Pre M = 10.02 (±3.29); *n* = 128; Post-intervention M = 8.12(±3.33); *p* = 0.0001
Chiang et al. 2012	Burden: 28 items Caregiver Burden Inventory (CBI)	Baseline, end of the programme (1 month)	T0 CG M = 41.50 (±10.12); *n* = 30 T0 IG M = 43.93 (±12.39); *n* = 30; *p* = 0.41 T1 CG M = 32.37 (±9.15); *n* = 30 T1 IG M = 23.27 (±10.91); *n* = 30; *p* < 0.001

CG, control group; IG, intervention group; WLCG, waiting list group; M, median; ± SD.

### 3.6 Effects of intervention by technologies

The efficacy of the interventions is also presented in Table 4. Among the thirteen studies that assessed caregiver burden, only three did not report statistically significant results (23, 24, 35). Of the six studies that assessed stress, only one reported no statistically significant results associated with the intervention (38). Fewer studies examined the impact of interventions on overall quality of life (28, 32) mental health (29, 30), and well-being (24, 37), which limits the ability to draw conclusions regarding these variables. Nevertheless, Minaei-Moghadam et al. (32) reported a significant improvement in quality-of-life scores for the intervention group compared to the control group. Three studies reported improvements in mental health in caregivers receiving interventions (28–30), although no statistically significant differences were observed in physical health (28). Fuller-Tyszkiewicz et al. (37) documented a statistically significant decline in subjective well-being among participants in the control group, a trend not observed in the intervention group. Similarly, Meichsner et al. (24) reported significant improvements in emotional well-being over time, with most participants showing positive changes.

### 3.7 Risk of bias

The bias analysis was performed separately for RCT and quasi- experimental studies.

Table 5 presents a methodological quality analysis of the selected RCTs. The studies exhibit overall strong methodological quality, with scores ranging from 7/13 to 11/13 for RCTs. All these studies employed true randomization, ensuring equitable distribution of participants and consistency in outcome measurement and statistical analysis. Notwithstanding these results, the analysis had an important limitation because of the lack of allocation concealment and blinding, particularly among participants, intervention providers, and outcome assessors. These aspects increased the risk of performance and detection bias, potentially impacting the internal validity of the study findings. Furthermore, some RCTs did not fully report participant follow-up, potentially affecting the reliability of their results. The studies that scored the highest (28, 30, 38) demonstrated greater adherence to RCT design principles while some studies with lower scores (24, 35) lacked key methodological weaknesses.

**Table 5:** Methodological quality analysis of selected RCT studies.

Author/year	Rahimi et al. 2025	Kabotari et al. 2025	Ganefianty et al. 2024	Minaei-Moghadam et al. 2024	Riegel et al. 2024	Douglas et al., 2023	Tinoco-Camarena et al. 2023	Hepburn et a. 2022	Ferré-Grau1 et al. 20221	Namjoo et al. 2021	Fuller-Tyszkiewicz et al. 2020	Possin et al. 2019	Meichsner et al. 2019	Applebaum et al. 2018
Was true randomization used to assign the participants to treatment groups?	y	Y	y	Y	Y	Y	Y	Y	Y	Y	Y	Y	Y	Y
Was allocation to treatment groups concealed?	y	U	y	Y	Y	Y	Y	U	U	U	U	U	N	U
Were treatment groups similar at the baseline?	y	Y	y	Y	Y	Y	Y	Y	Y	Y	Y	Y	N	Y
Were participants blind to treatment assignment?	y	U	U	N	N	U	Y	U	N	U	Y	N	N	N
Were those delivering treatment blind to treatment assignment?	N	N	U	N	N	N	N	N	N	N	N	N	N	N
Were outcomes assessors blind to treatment assignment?	Y	U	N	Y	Y	U	Y	Y	N	U	U	Y	N	U
Were treatment groups treated identically other than the intervention of interest?	Y	y	Y	Y	Y	Y	Y	Y	Y	Y	Y	Y	Y	Y
Was follow-up complete and if not, were differences between groups in terms of their follow-up adequately described and analysed?	N	Y	Y	U	Y	Y	U	Y	N	Y	U	Y	Y	N
Were participants analysed in the groups to which they were randomized?	Y	Y	y	Y	Y	Y	Y	Y	Y	Y	Y	Y	Y	Y
Were outcomes measured in the same way for treatment groups?	Y	Y	Y	Y	Y	Y	Y	Y	Y	Y	Y	Y	Y	Y
Were outcomes measured in a reliable way?	Y	Y	Y	Y	Y	Y	Y	Y	Y	Y	Y	Y	Y	Y
Was appropriate statistical analysis used?	Y	Y	Y	Y	Y	Y	Y	Y	Y	Y	Y	Y	Y	Y
Was the trial design appropriate, and any deviations accounted for in the conduct and analysis of the trial?	Y	Y	Y	Y	Y	Y	Y	Y	Y	Y	Y	Y	Y	Y
Score	11/13	9/13	10/13	10/13	11/13	10/13	11/13	10/13	8/13 13	9/13 13	9/13 13	10/13	8/13 13	7/13 13

Y, yes; N, no; U, undetermined.

Table 6 presents a methodological quality analysis of the two included experimental studies that were not RCTs. Overall, the studies demonstrated moderate to strong methodological foundations, with scores of 6/9 and 9/9. Both quasi-experimental studies clearly established causal relationships between variables, ensuring the direction of the effect was well defined. However, Easom et al. (36) lacked a control group, limiting its ability to establish causality, and variations in treatment conditions introduced potential confounders.

**Table 6:** Methodological quality analysis of the other experimental selected studies.

Author/year	Easom et al. 2018	Chiang et al. 2012
Is it clear in the study what the “cause” and what the “effect” is (i.e., there is no confusion about which variable comes first)?	Y	Y
Was there a control group?	N	Y
Were participants included in any comparisons similar?	Y	Y
Were the participants included in any comparisons receiving similar treatment/care other than the exposure or intervention of interest?	N	Y
Were there multiple outcome measurements, both pre- and post-intervention/exposure?	Y	Y
Were the outcomes of participants included in any comparisons measured in the same way?	Y	Y
Were outcomes measured in a reliable way?	Y	Y
Was follow-up complete, and if not, were differences between groups in terms of their follow-up adequately described and analysed?	N	Y
Was appropriate statistical analysis used?	Y	Y
Score	6/9	9/9

Y, yes; N, no; U, undetermined.

### 3.8 Effects of digital technology-based interventions

In the meta-analysis, caregiver burden was evaluated as an outcome at both 3-month and 6-month follow-up, whereas stress was assessed only at 3-month follow-up.

#### 3.8.1 Burden at <3 months

**Figure 3:**
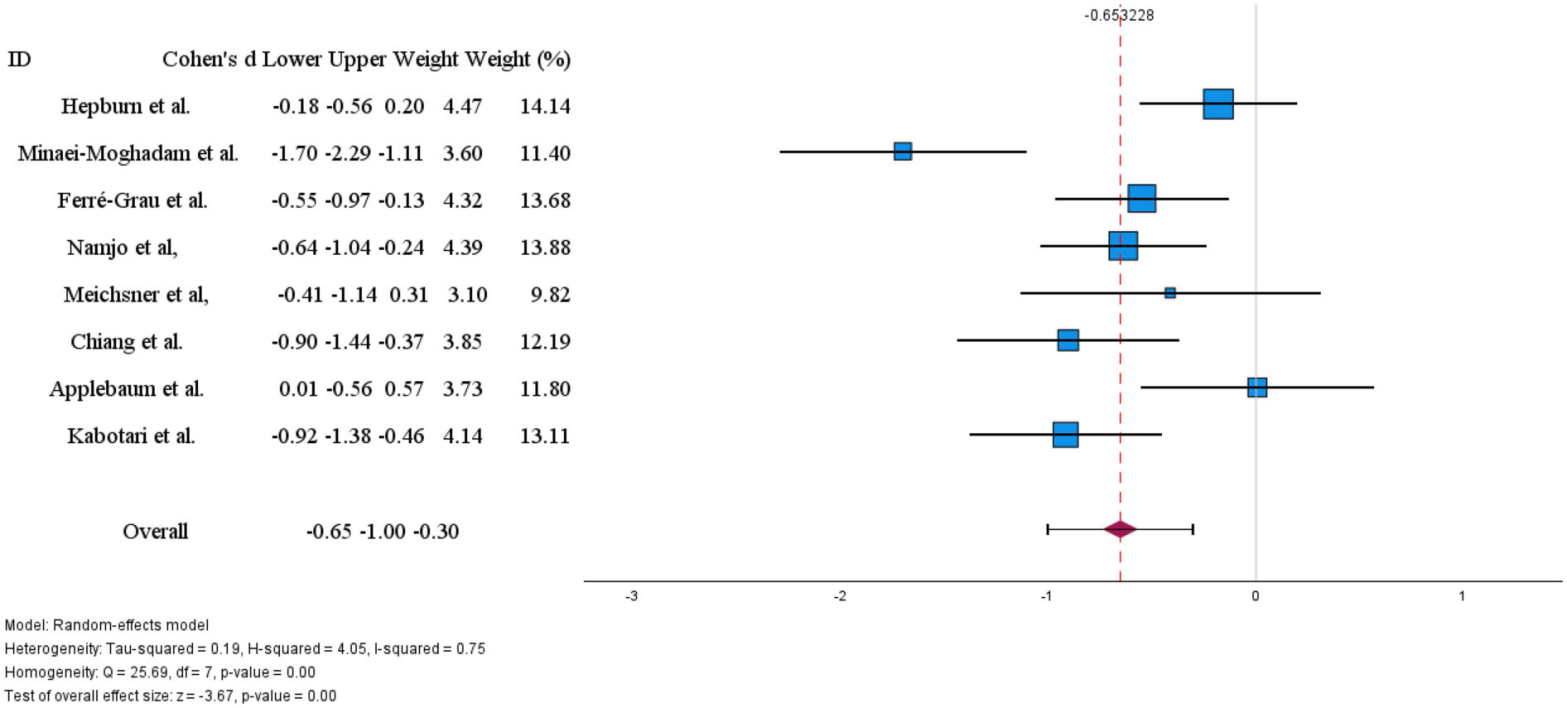
Effects of digital technology-based interventions on caregiver burden in a period of less than 3 months.

The meta-analysis initially included nine studies. Using a random-effects model, the overall effect size was Cohen's d = −1.02 (95% CI: −1.80 to −0.24; *p* = 0.01). Three studies reported no statistically significant differences in burden (23, 24, 35). This indicates a significant negative effect of large magnitude. However, heterogeneity across studies was extremely high [Q(8) = 93.78, *p* < 0.001; I^2^ = 95%], suggesting that much of the observed variation was due to substantial differences between studies rather than chance. Inspection of the forest plot revealed that the study by Ganefianty et al. (31) reported an unusually large effect size (d = −4.09), which could be considered an outlier. To assess the robustness of the findings, a sensitivity analysis was conducted excluding Ganefianty et al. In this revised analysis, based on eight studies (Figure 3), the overall effect remained significant, with Cohen's d = −0.65 (95% CI: −1.00 to −0.30; *p* < 0.01). Although the effect size was reduced, it still indicated a moderate negative impact. Heterogeneity decreased [Q(7) = 25.69, *p* < 0.01; I^2^ = 75%] but remained substantial, suggesting that the outlier had considerably inflated inconsistency among the results. Overall, both the initial and sensitivity analyses demonstrate a significant negative effect. The exclusion of the outlier produced a more conservative yet robust estimate, strengthening the reliability of the findings.

#### 3.8.2 Burden at 6 months

Only two studies (23, 24) reported caregiver burden at six-month follow-up. Given the limited number of studies, we did not conduct a meta-analysis. Hepburn et al. (23) found a small, non-significant reduction in burden (Cohen's d = −0.13), while Meichsner et al. (24) reported a non-significant increase (Cohen's d = 0.53). The conflicting direction of effects and small number of studies preclude definitive conclusions about six-month effectiveness.

#### 3.8.3 Stress at <3 months

**Figure 4:**
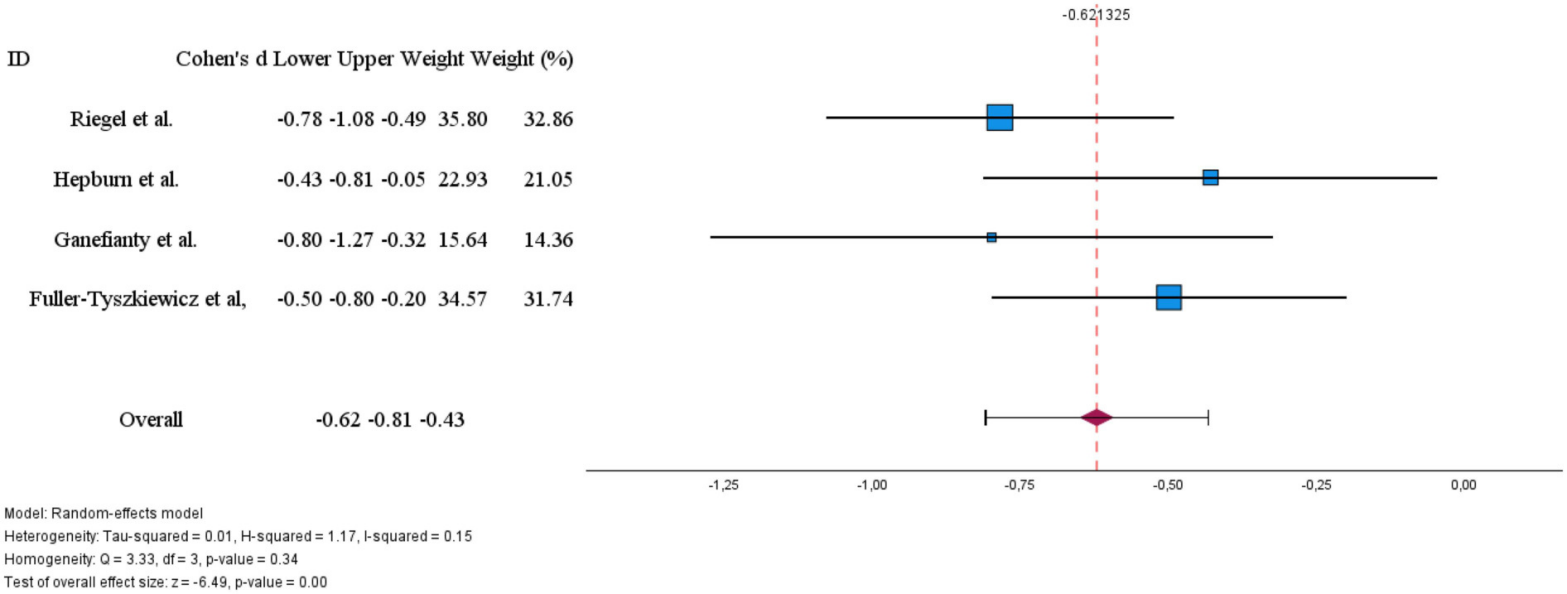
Effects of digital technology-based interventions on caregiver stress in a period of less than 3 months.

Four studies provided sufficient data to assess the effectiveness of technology-based interventions in reducing caregiver stress (Figure 4). The pooled effect size was Cohen's d = −0.62 (95% CI:−0.81 to −0.43, *p* < 0.01), indicating a moderate and statistically significant reduction in stress favouring the intervention.

Individual study effects ranged from d = −0.43 to d = −0.80, with all confidence intervals excluding zero, demonstrating consistency in the direction of effect across studies. Heterogeneity was low [I^2^ = 15%; Q(3) = 3.33, *p* = 0.34], suggesting minimal variability between studies and supporting the reliability of the pooled estimate.

#### 3.8.4 Quality of life and well-being

Six studies assessed quality of life and/or well-being as outcomes (24, 28–30, 32, 37). Two studies (24, 30) did not report sufficient statistical data for inclusion in the meta-analysis, limiting the pooled analysis to four studies.

**Figure 5:**
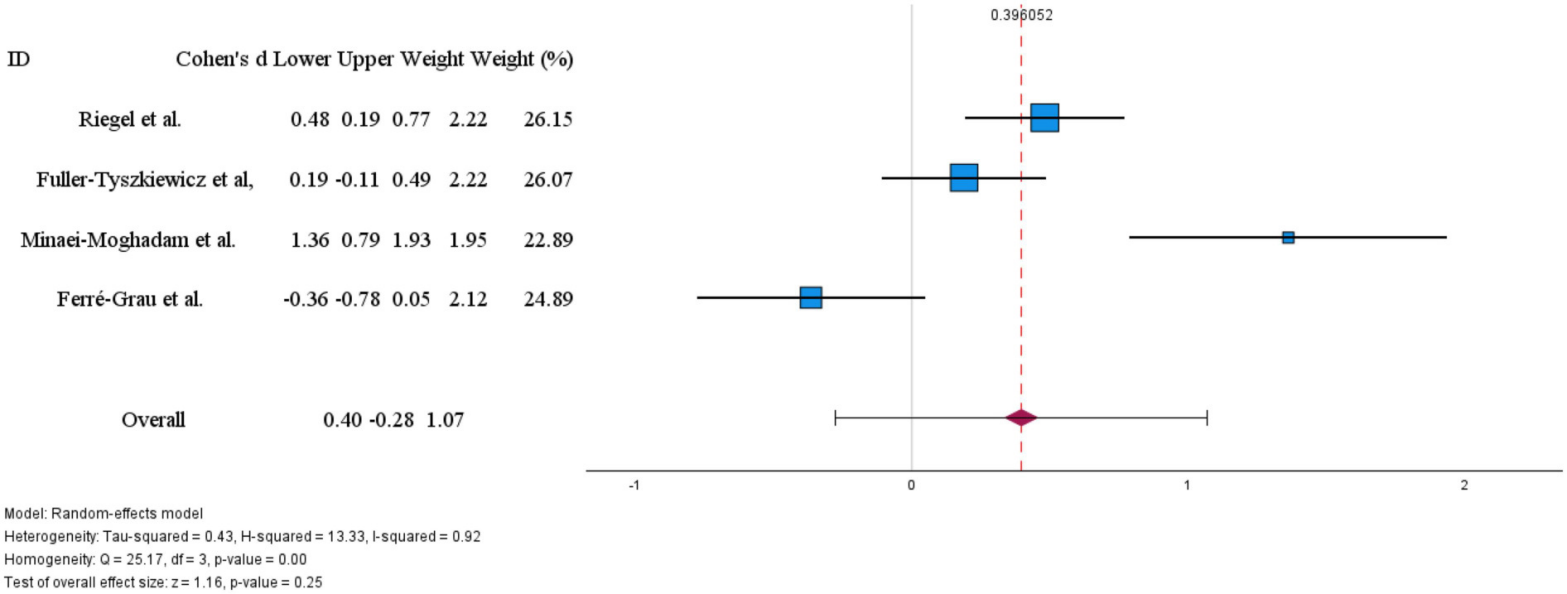
Effects of digital technology-based interventions on the quality of life in a period of less than 3 months.

The meta-analysis (Figure 5) revealed a small, non-significant overall effect favouring the intervention (Cohen's d = 0.40, 95% CI: −0.28 to 1.07, *p* = 0.25). Notably, heterogeneity was very high [I^2^ = 92%; Q(3) = 25.17, *p* < 0.01], indicating substantial and statistically significant variability across studies. This heterogeneity is reflected in the divergent findings: while Riegel et al. (28) found a moderate improvement in quality of life (d = 0.48 for mental health SF36 subscale), Minaei-Moghadam et al. (32) reported a high improvements in quality of life (d = 1.36), Fuller-Tyszkiewicz et al. (37) found a small, non-significant effect (d = 0.19), and Ferré-Grau et al. (29) reported a small effect in the opposite direction (d = −0.36).

The marked heterogeneity and inconsistent direction of effects preclude a definitive conclusion about the effectiveness of technology-based interventions on caregiver quality of life. These divergent results may reflect important differences in intervention characteristics (e.g., type, intensity, delivery mode), caregiver populations, disease stages, or outcome measurement instruments, suggesting that effectiveness may be context-dependent and warranting further investigation through subgroup analyses or qualitative synthesis.

## 4 Discussion

This systematic review and meta-analysis aimed to assess how effective digital technology-based interventions are at reducing the burden and stress experienced by family caregivers. Our findings suggest that these interventions, especially those focused on psychoeducation, show promise. However, their effectiveness is shaped by several factors that need closer examination.

Our review of 16 studies highlighted a variety of technological approaches. These included mobile applications, websites, and tele-coaching, used to support caregivers looking after individuals with various health conditions such as dementia, heart failure, and cancer. Most caregivers in these studies were women, typically partners or daughters of the care recipients.

The meta-analysis showed a significant overall short-term effect (less than 3 months) in reducing caregiver burden (Cohen's d = −0.65) and stress (d = −0.62). The impact on stress was consistent across studies, with low variability. However, the effect on caregiver burden showed considerable variability (I^2^ = 75%), even after removing an outlier study.

This suggests that the effectiveness of these interventions may vary considerably depending on the context and research methods employed. In contrast, no statistically significant effect was observed on caregivers' quality of life. This outcome was characterised by very high variability (I^2^ = 92%) and inconsistent findings across studies. Firm conclusions regarding long-term effectiveness could not be drawn due to the limited number of studies with six-month follow-ups and their conflicting results.

Our findings largely align with existing literature, which supports the effectiveness of technology-based interventions in reducing caregiver burden (13–17, 41).

A recent systematic review further reinforces this, providing a moderate estimate of effect for short-term reductions in burden and stress (17).

However, our analysis highlights the complexity behind these effects. The high variability observed in caregiver burden suggests that the effectiveness of interventions is not uniform. Factors such as the intervention's delivery method (synchronous vs. asynchronous), intensity, duration, and the type of professional support can all influence the outcomes. This point has also been raised by other researchers (15, 42, 43). Our observation that synchronous approaches might be better suited for psychological outcomes, such as stress, while asynchronous methods could be more useful for self-care and disease management, adds an important nuance to this discussion.

Furthermore, the lack of a clear effect on quality of life, which is often a secondary outcome in studies, contrasts with improvements seen in related areas like self-efficacy and reduced depressive symptoms global (16, 41, 44). This suggests that the impact of these interventions might be more specific rather than broad.

### 4.1 Strengths and limitations

One of the main strengths of this study is its rigorous methodology, which followed JBI and PRISMA guidelines, and the meta-analysis that allowed us to quantify the effectiveness of the interventions. However, several limitations should be considered when interpreting the results.

The primary limitation is the high methodological variability among the included studies. Interventions differed considerably in terms of technological approach, frequency, duration, and content. Additionally, a wide range of tools were used to measure the same outcomes, making direct comparisons and generalisation of results difficult. The nature of the control groups also varied, from usual care to waiting lists or partial access to the intervention, which could have influenced the size of the observed effects.

Another significant limitation is the lack of long-term evaluation. Most studies assessed effects only at the end of the intervention, and the scarcity of follow-up data prevented us from determining if the benefits lasted over time. This is a critical gap, as sustained effects are essential for adherence and the lasting impact of such programmes.

Regarding the methodological quality of the primary studies, our analysis of bias risk revealed important weaknesses. Specifically, most randomised controlled trials lacked proper allocation concealment and blinding of participants, professionals, and outcome assessors. These factors increase the risk of performance and detection bias, respectively, potentially compromising the internal validity of the results. Finally, the exclusion of some studies from the meta-analysis due to insufficient statistical data (means and standard deviations) might have limited the precision of our effect estimates.

### 4.2 Clinical implications

The findings of this study have direct implications for clinical practice, particularly for nurses, who were most frequently involved in delivering these interventions (45–48). The evidence shows that digital interventions can be an effective tool for reducing caregiver burden and stress in the short term. Therefore, healthcare professionals should consider integrating these technologies as a complement to usual care, offering more accessible and continuous support.

The choice of technological modality requires careful consideration. Our results suggest that the approach (synchronous, asynchronous, or mixed) can influence outcomes, with real-time interaction (synchronous) potentially being more beneficial for emotional and psychological support (25, 28, 33, 36). Interventions should be tailored to the specific needs of the caregiver, taking into account their goals, digital literacy, and the condition of the person being cared for (17). The central role of nurses and multidisciplinary teams, identified in the studies, highlights the importance of a collaborative and holistic approach to supporting caregivers, using technology to extend the reach and effectiveness of professional care.

### 4.3 Implications for future research

The limitations identified in this review point to several directions for future research. Firstly, it is crucial to conduct studies with longer follow-up periods to assess how sustainable the effects of interventions are in the medium and long term. While short-term effectiveness has been demonstrated, it is unclear whether the benefits persist over time. Secondly, the high variability in outcomes, especially for caregiver burden and quality of life, highlights the need to investigate the most effective “active ingredients’ of these interventions. Future studies should compare different modalities (e.g., synchronous vs. asynchronous), durations, and intensities of intervention to determine which combinations produce the best results for different caregiver profiles.

To allow for more robust comparisons between studies and more precise meta-analyses, greater standardisation of outcome assessment tools is essential. The use of a core outcome set for caregiver burden, stress, and quality of life would be a significant advancement for the field. Additionally, the methodological quality of clinical trials needs improvement, with particular attention to the proper implementation and reporting of allocation concealment and blinding, to minimise bias risks. Finally, given the inconclusive results, caregivers' quality of life and well-being deserve to be investigated as primary outcomes in future studies.

## 5 Conclusions

In conclusion, this systematic review and meta-analysis confirms the potential of digital technology-based interventions as an effective strategy to reduce family caregiver burden and stress in the short term. However, the effectiveness of these interventions is not universal; it is influenced by various methodological and contextual factors, leading to considerable variability in effects, particularly concerning caregiver burden and quality of life. The long-term sustainability of these benefits remains an open question. For the full potential of these technologies to be realised, future research should focus on optimising intervention protocols, improving the methodological quality of studies, and standardising outcomes. This will enable the development of more robust, personalised, and lasting support strategies for family caregivers.

## Data Availability

The original contributions presented in the study are included in the article/Supplementary Material, further inquiries can be directed to the corresponding author/s.
